# Cochlear Size Assessment Predicts Scala Tympani Volume and Electrode Insertion Force- Implications in Robotic Assisted Cochlear Implant Surgery

**DOI:** 10.3389/fsurg.2021.723897

**Published:** 2021-09-30

**Authors:** Anandhan Dhanasingh, Chloe Swords, Manohar Bance, Vincent Van Rompaey, Paul Van de Heyning

**Affiliations:** ^1^Research and Development Department, MED-EL, Innsbruck, Austria; ^2^Department of Translational Neurosciences, Faculty of Medicine and Health Sciences, University of Antwerp, Antwerp, Belgium; ^3^Department of Physiology, Development and Neurosciences, University of Cambridge, Cambridge, United Kingdom; ^4^Department of Clinical Neurosciences, University of Cambridge, Cambridge, United Kingdom; ^5^Department of Otorhinolaryngology and Head & Neck Surgery, Antwerp University Hospital, Antwerp, Belgium

**Keywords:** scala tympani volume, cochlear size, electrode insertion speed, electrode insertion force, robot assisted surgery

## Abstract

**Objectives:** The primary aim was to measure the volume of the scala tympani (ST) and the length of the straight portion of the cochlear basal turn from micro-computed tomography (μCT) images. The secondary aim was to estimate the electrode insertion force based on cochlear size and insertion speed. Both of these objectives have a direct clinical relevance in robotic assisted cochlear implant (CI) surgery.

**Methods:** The ST was segmented in thirty μCT datasets to create a three-dimensional (3D) model and calculate the ST volume. The diameter (A-value), the width (B-value), and the straight portion of the cochlear basal turn (S-value) were measured from the oblique coronal plane. Electrode insertion force was measured in ST models of two different sizes, by inserting FLEX24 (24 mm) and FLEX28 (28 mm) electrode arrays at five different speeds (0.1, 0.5, 1, 2, and 4 mm/s).

**Results:** The mean A-, B-, and S-values measured from the 30 μCT datasets were 9.0 ± 0.5, 6.7 ± 0.4, and 6.9 mm ± 0.5, respectively. The mean ST volume was 34.2 μl ± 7 (range 23–50 μl). The ST volume increased linearly with an increase in A- and B-values (Pearson's coefficient *r* = 0.55 and 0.56, respectively). The A-value exhibited linear positive correlation with the B-value and S-value (Pearson's coefficient *r* = 0.64 and *r* = 0.66, respectively). In the smaller of the two ST models, insertion forces were higher across the range of insertion speeds during both array insertions, when compared to the upscaled model. Before the maximum electrode insertion depths, a trend toward lower insertion force for lower insertion speed and vice-versa was observed.

**Conclusion:** It is important to determine pre-operative cochlear size as this seems to have an effect upon electrode insertion forces. Higher insertion forces were seen in a smaller sized ST model across two electrode array lengths, as compared to an upscaled larger model. The ST volume, which cannot be visualized on clinical CT, correlates with clinical cochlear parameters. This enabled the creation of an equation capable of predicting ST volume utilizing A- and B-values, thus enabling pre-operative prediction of ST volume.

## Introduction

Cochlear implant (CI) technology has evolved over the last 40 years reaching its maturity in terms of basic technological advancements (1). Throughout the years, surgical importance has been placed on the optimal placement of the CI electrode array in order to preserve the intra-cochlear structures (2). The steps in CI surgery, including cortical mastoidectomy, posterior tympanotomy, round window (RW) opening and the electrode array insertion, are performed manually. As a result, the hearing outcomes of CI recipients may be influenced by the surgical learning curve of every CI surgeon (3).

Intra-cochlear structures are delicate. Ishii et al. reported that electrode array insertion force above 35 milli-newtons (mN) would result in disturbance of the basilar membrane (4). The ability of humans to manually respond to minute changes in insertion forces in the range of 35 mN may be less reliable when compared to the haptic feedback systems available with automated insertion systems. In addition, it has been reported that electrode insertion speed has an influence on structure and hearing preservation (5). Thus, as research interests turn toward automated and robotic-assisted electrode insertion, the quantification of the optimal insertion speed that offers the minimum insertion force along with the ability to insert the electrode fully inside the cochlea would be beneficial. During the insertion process, the tip of an electrode array is angulated toward the lateral wall at the end of the straight portion of cochlear basal turn and is likely to collide with this. Appreciating how this straight portion length varies with the overall variation in other cochlear parameters, such as scala tympani (ST) volume, would enable automated adjustment of insertion speeds when approaching the end of the cochlear basal turn.

Personalized treatment in cochlear implantation is being developed at multiple timepoints throughout the patient's journey, for instance during audio processor fitting (6) and otological pre-planning software (e.g., OTOPLAN®) in assessing cochlear size and choosing electrode array length matching the cochlear size (7). Robotic assisted CI surgery and controlled speed electrode array insertion, such as the HEARO® system and ROBOTOL®, respectively (8–10) are in the early stages of clinical practice. To complement these technological advancements and to take the concept of personalized CI treatment to the next level where the electrode insertion speed can be personalized to the individual's ST size and volume, a study on the following objectives is essential. Estimating the ST volume and length of the straight portion of cochlear basal turn based on pre-operative assessment of cochlear parameters. Studying the changes in electrode array insertion force from *in-vitro* insertion experiments in different sized ST models with varying volumes, employing different electrode array lengths inserted with different insertion speeds.

Therefore, the primary aim of this study was to measure the volume of the ST from micro-computed tomography (μCT) images and establish whether there is a relationship with basic cochlear parameters [length of straight portion of the basal turn (S-value), basal turn diameter (A-value) and cochlear width (B-value)]. This would help in the estimation of ST volume from the pre-operative clinical images. The secondary aim was to study the electrode array insertion forces of two variants of FLEX electrodes (FLEX28 and FLEX24), inserted at various insertion speeds in two different sized ST plastic models. This would allow us to understand how the electrode insertion forces changes with the changes in the ST volume indirectly estimated from the cochlear parameters. ST fluid itself would act as an impedance to electrode insertion and therefore the knowing the ST volume is of clinical interest. All this pre-operative information will add to the wealth of information available to the operating surgeon to potentially influence intra-operative behavior.

## Materials and Methods

### Image Analysis

**Figure 1:**
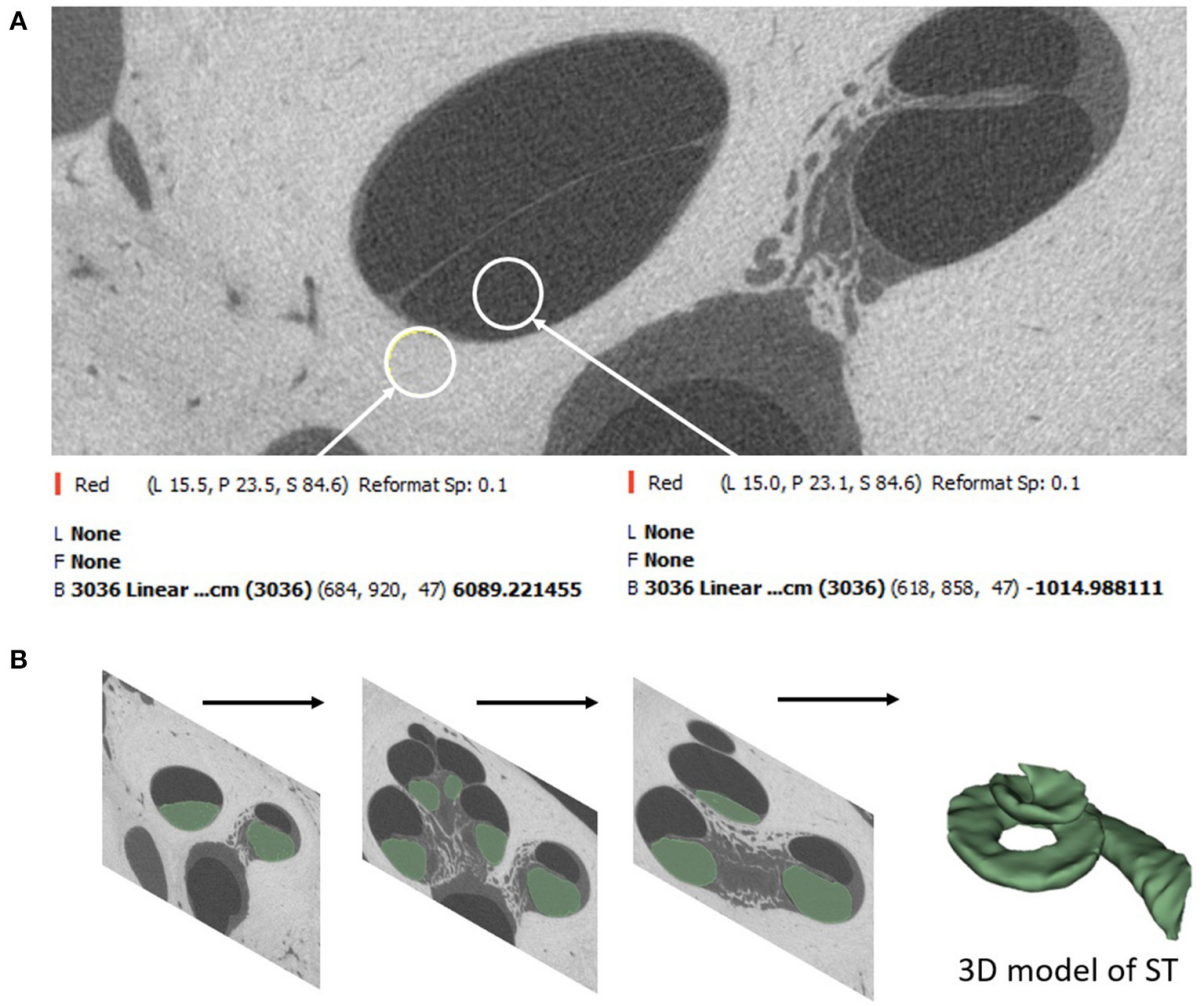
Segmentation of ST starts by setting a tight grayscale threshold that distinguishes between the fluid filled ST and the surrounding bony region **(A)**. Segmentation is done by shading the areas of interest from all image slices **(B)**.

Image analysis was performed on thirty μCT image datasets of cadaveric temporal bones. There were no inner ear malformations present. Fifteen raw datasets were sourced from the HEAR-EU project (https://cordis.europa.eu/project/id/304857), and the other fifteen raw datasets were from Cambridge (CS and MB). The μCT images [24–30 micron (μ) isotropic voxel-sizes] were analyzed using Slicer Version 4.10.2 (https://www.slicer.org/) in the HEAR-EU data, and Stradview (Version 6.1) for the Cambridge data. The reason for using two different image analysis software was due to the availability of the specific software at MED-EL Innsbruck and University of Cambridge, Cambridge, respectively. Three-dimensional (3D) segmentation of the ST from these combined raw datasets was performed in this study following the steps described by Dhanasingh et al. (11). In brief, the image datasets were loaded into two different 3D segmentation software. Segmentation of the ST was performed as precisely as possible in the axial plane in every slice of the cochlea by setting tight grayscale threshold to avoid capturing undesired structures (Figure 1A). Grayscale thresholding to capture the desired structures was done individually for every individual image data set. Figure 1B shows an example of grayscale thresholding; the grayscale of the otic capsule is 6,089 (bright = bone) and the grayscale of membranous labyrinth (dark = labyrinth) is −1,014, thus setting the thresholding −1,014 and 6,089 for this temporal bone. The volume of ST was measured using the command “segment statistics.”

**Figure 2:**
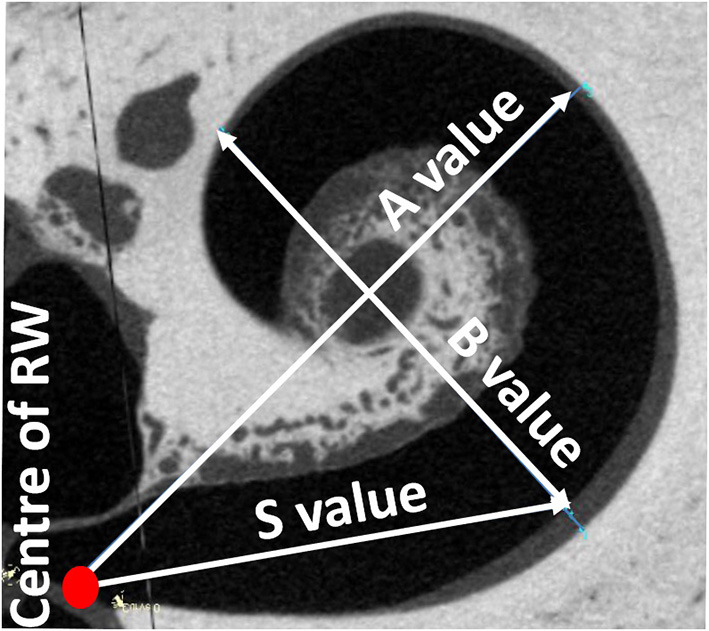
Oblique coronal plane through the basal turn with the cochlear parameters defined.

The A-value was measured in the oblique coronal plane starting from the center of the round window membrane (RWM) and passing through the centre of the cochlea to the opposite lateral wall, as originally described by Escude et al. (12) (Figure 2). The B-value refers to the width of the basal turn which is measured by drawing a line perpendicular to the A-value. The S-value refers to the length of the straight portion of the cochlear basal turn starting from the RWM to the inferior end of the B-value line. Measurements were cross-checked and validated by two authors.

### Electrode Insertion Force Measurement

**Figure 3:**
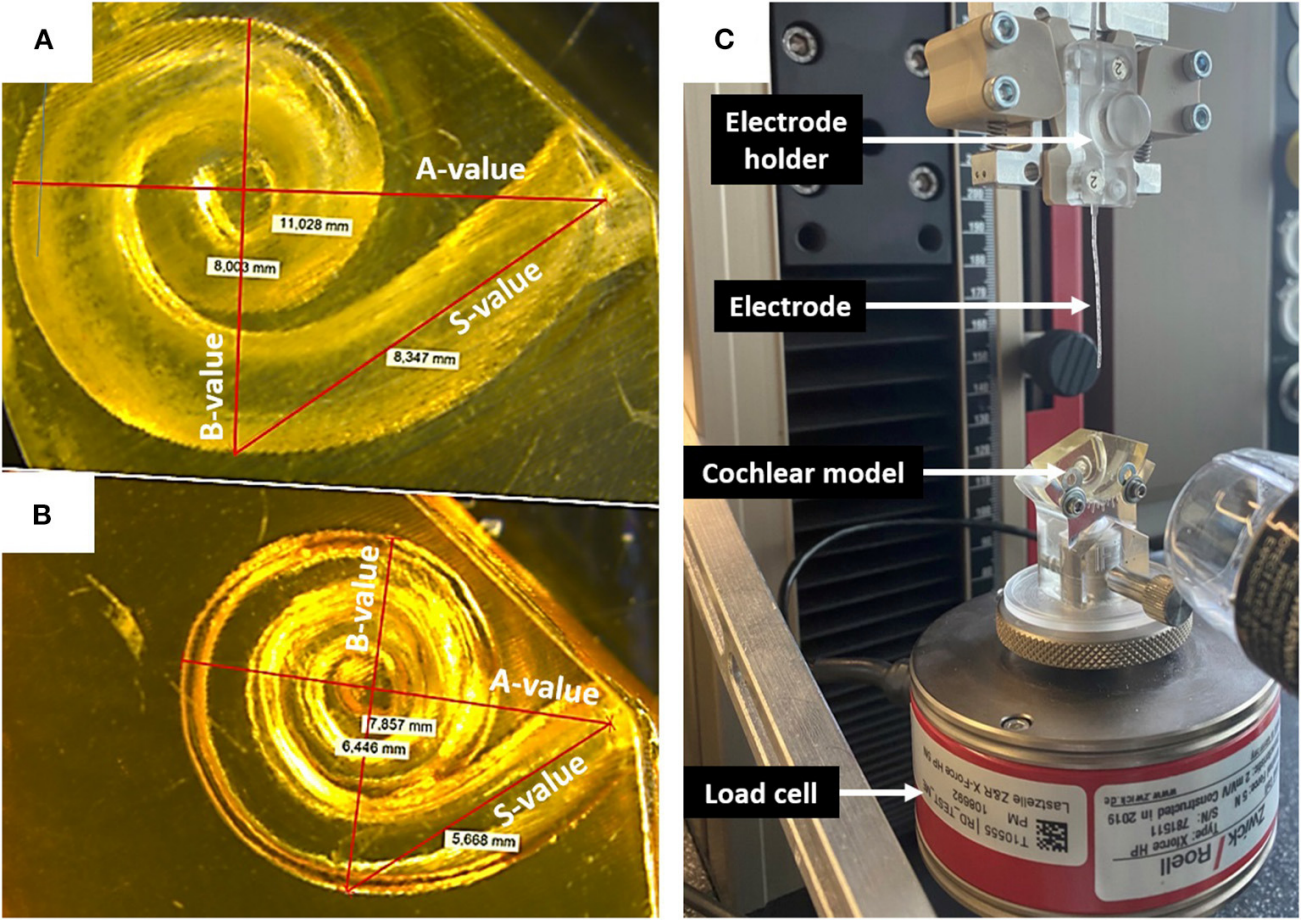
**(A)** Upscaled ST model: A-value 11 mm; B-value 8 mm; S-value 8.3 mm. **(B)** Small ST model: A-value 7.9 mm; B-value 6.4; S-value 5.6 mm. **(C)** Electrode insertion force measurement test set-up.

All electrode insertion experiments were performed in an acrylic 3D model of the ST in two different sizes (Figures 3A,B). The fabrication process is described by Leon et al. (13). The volume of the smaller model was 35 μl; whereas the larger model had the same morphology but was upscaled by 1.5 × to 52.5 μl. This enabled direct comparison of the impact of size, irrespective of confounding anatomical variations. Insertion forces were measured using a commercially available S-shaped, single axis (compression and tension) load cell with a measuring range up to 5 newtons (N) (Zwick Roell, Xforce HP https://www.zwickroell.com/accessories/xforce-load-cells/). The ST model was placed atop the load cell and filled with 0.1% soap solution to act as a lubricant. The sensor was mounted on a positioning device to enable the precise adjustment of the cochlear model's position and orientation with respect to insertion of the electrode array (Figure 3C). The experiment was performed across five insertion speeds (0.1, 0.5, 1, 2, and 4 mm/s) using two different electrode array lengths of 24 mm (FLEX24) and 28 mm (FLEX28) at full insertion. The volume of FLEX24 and FLEX28 electrode arrays were calculated as 6.9 and 8.8 μl, respectively. For each length of the electrode array, the measurements were repeated three times, plus repeated with three different arrays. Thus, nine measurements were performed in total per implant length for each insertion speed.

### Statistical Analysis

Regression estimates between the A- and B-values and A- and S-values (confidence level 0.95) were determined using the data analysis tool in STATISTICA software (version 14.0, https://www.tibco.com/products/data-science). Pearson's coefficient (r) was used to assess the strength of correlation between measurements. A multiple linear regression model was designed to formulate an equation that predicted the ST volume, using the A- and B-values. Two-way ANOVA test with replication was used to check the significance in electrode insertion forces comparing between the electrode length, ST models and the insertion speeds.

## Results

### Data Analysis

Table 1 summarizes the cochlear parameters as measured by A-, B-, and S- values, and ST volume from both HEAR-EU and Cambridge datasets. The mean A-, B-, and S-values of combined datasets were 9.0 ± 0.5, 6.7± 0.4, and 6.9 ± 0.5 mm, respectively. The mean ST volume was 34.2 ± 7 μl although this value varied significantly between different samples (range 23–50 μl). The predicted ST volume was calculated applying Equation 1, applying the A-and the B-values, as described below.

**Table 1:** A-, B-, S-values measured from the μCT images in the oblique coronal view of cochlear basal turn and ST volume measured from 3D segmented model of ST.

**Source of μCT datasets**	**A-value (mm)**	**B-value (mm)**	**S-value (mm)**	**Measured ST volume (μl)**	**Predicted ST volume (μl)**	**Absolute error (%)**
HEAR-EU (*n* = 15)	9.4	6.8	7.7	36	36.8	2.2
	8.7	6.2	6.8	33	29.7	11
	8.9	7.1	7.0	45	36.1	24.7
	8.4	6.7	6.6	33	31.2	5.6
	9.7	6.9	7.9	49	38.9	26
	8.7	6.6	6.9	34	32.2	5.7
	9.6	6.9	7.5	50	38.4	30.3
	8.6	7.0	7.1	46	34	35.4
	8.7	6.1	6.9	28	29.2	4.2
	8.6	6.0	7.1	28	28.2	0.55
	8.8	6.5	7.5	28	32.1	12.7
	9.2	6.8	7.2	35	35.81	2.3
	8.8	6.2	6.7	29	30.3	4.3
	8.7	6.5	7.0	34	32	7.7
	9.3	6.0	7.0	43	37	17.6
Cambridge (*n* = 15)	9.7	7.2	6.9	36	41	11.4
	8.0	6.2	5.8	23	26.3	12.5
	8.8	6.4	6.0	29	31.3	7.4
	9.0	7.0	6.6	31	35.5	12.8
	8.5	6.6	6.3	27	31	12.8
	8.7	6.0	6.3	23	28.4	19
	9.5	7.0	7.1	36	38.4	6.3
	9.1	7.4	6.3	34	38.7	12.2
	9.1	7.3	6.8	32	38	15.7
	9.4	7.1	7.0	41	38.8	5.7
	9.2	7.0	6.9	36	36.9	2.4
	9.1	6.4	6.7	30	32.4	7.4
	9.5	7.4	7.4	31	41	24.3
	8.1	6.5	6.3	32	28.6	11.9
	9.5	7.1	6.9	34	39.4	13.6
Range	8.0–9.7	6.0–7.4	5.8–7.9	23–50	26.2–40.9	0.55–35.4
Mean +/- standard deviation	9.0 ± 0.5	6.7 ± 0.4	6.9 ± 0.5	34.2 ± 7	34.2 ± 4.2	12.2 ± 8.7

**Figure 4:**
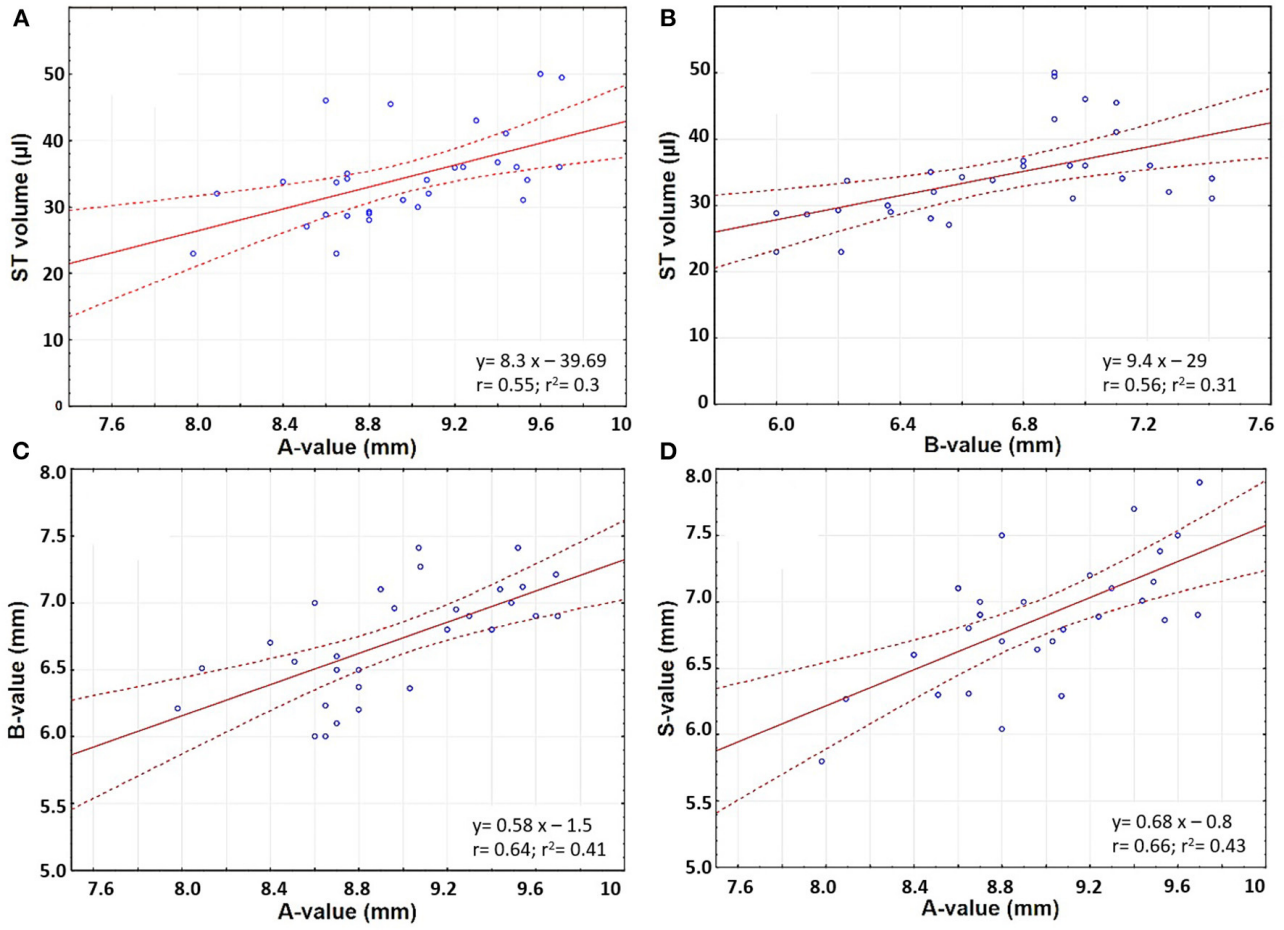
Scatter plots comparing **(A)** A-value and ST volume; **(B)** B-value and ST volume; **(C)** A- and B- values; and **(D)** A- and S-values.

The ST volume increased linearly with an increase in A- and B-values (Pearson's coefficient r 0.55 and 0.56, respectively) (Figures 4A,B). The A-value correlated with the B- and S-values (Pearson's coefficient *r* 0.64 and 0.66, respectively) (Figures 4C,D).

### Prediction of ST Volume From Basic Cochlear Parameters

The multiple linear regression model to predict the ST volume from the A-, and the B-values resulted in the following equation (Equation 1). The A- and B-values were measured in mm.


$$\begin{array}{l} Equation\;1:\;Predicted\;ST\;volume\;\left(\mu l\right)=\left(A\;value^{*}5\right)\\ +\left(B\;value^{*}5.8\right)-49.7 \end{array}$$

The gray shaded columns in Table 1 corresponded to the predicted ST volume when applying Equation 1 and the error percentage between the estimated and measured ST volume. The mean measured and predicted ST volume was 34.2 μl in both cases, although the range varied from 23–50 to 26.2–40.0 μl, respectively.

### Electrode Insertion Force Measurement

**Figure 5:**
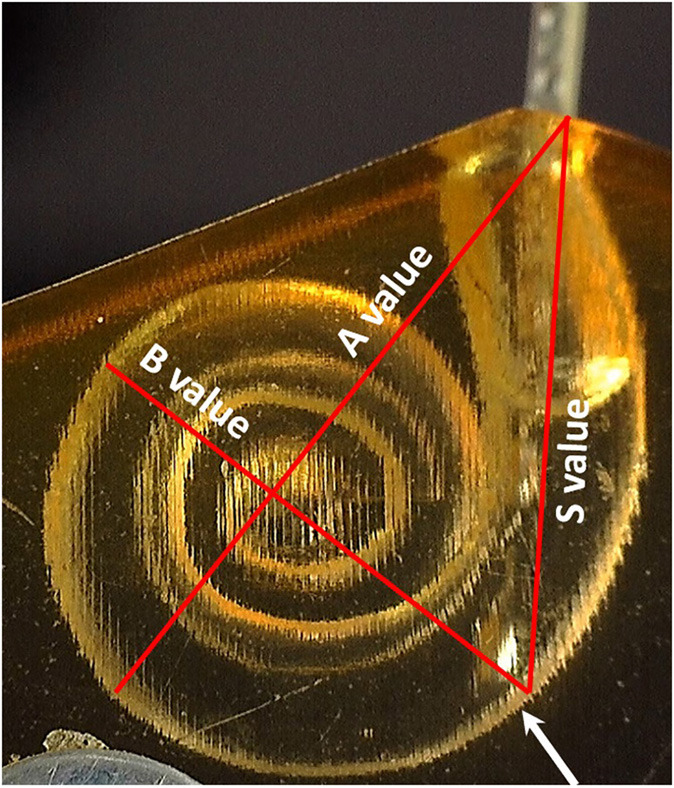
The point where the electrode tip touches the inner wall of the cochlear model corresponds to the end of the S-value as pointed by the white arrow.

As the electrode enters the cochlea, the tip touches the inner wall of the ST model at the S-value point (Figure 5).

The S-value was 5.6 mm for the smaller sized ST model and 8.3 mm for the upscaled model. The insertion force was almost negligible when measured up to these points for both array lengths and all insertion speeds (Table 2).

**Table 2:** Electrode insertion force (N) measured at the S-value point in the two different sized cochlear model.

	**Small sized model (S** **=** **5.6 mm)**					**Upscaled model (S** **=** **8.3 mm)**				
Insertion speed (mm/sec)	0.1	0.5	1	2	4	0.1	0.5	1	2	4
FLEX^28^	0	0.0004	0.0008	0.0001	0.0007	0	0.0003	0	0.0003	0.003
FLEX^24^	0	0.0003	0.0002	0.0004	0.001	0	0.0002	0.0001	0.0002	0.0005

**Figure 6:**
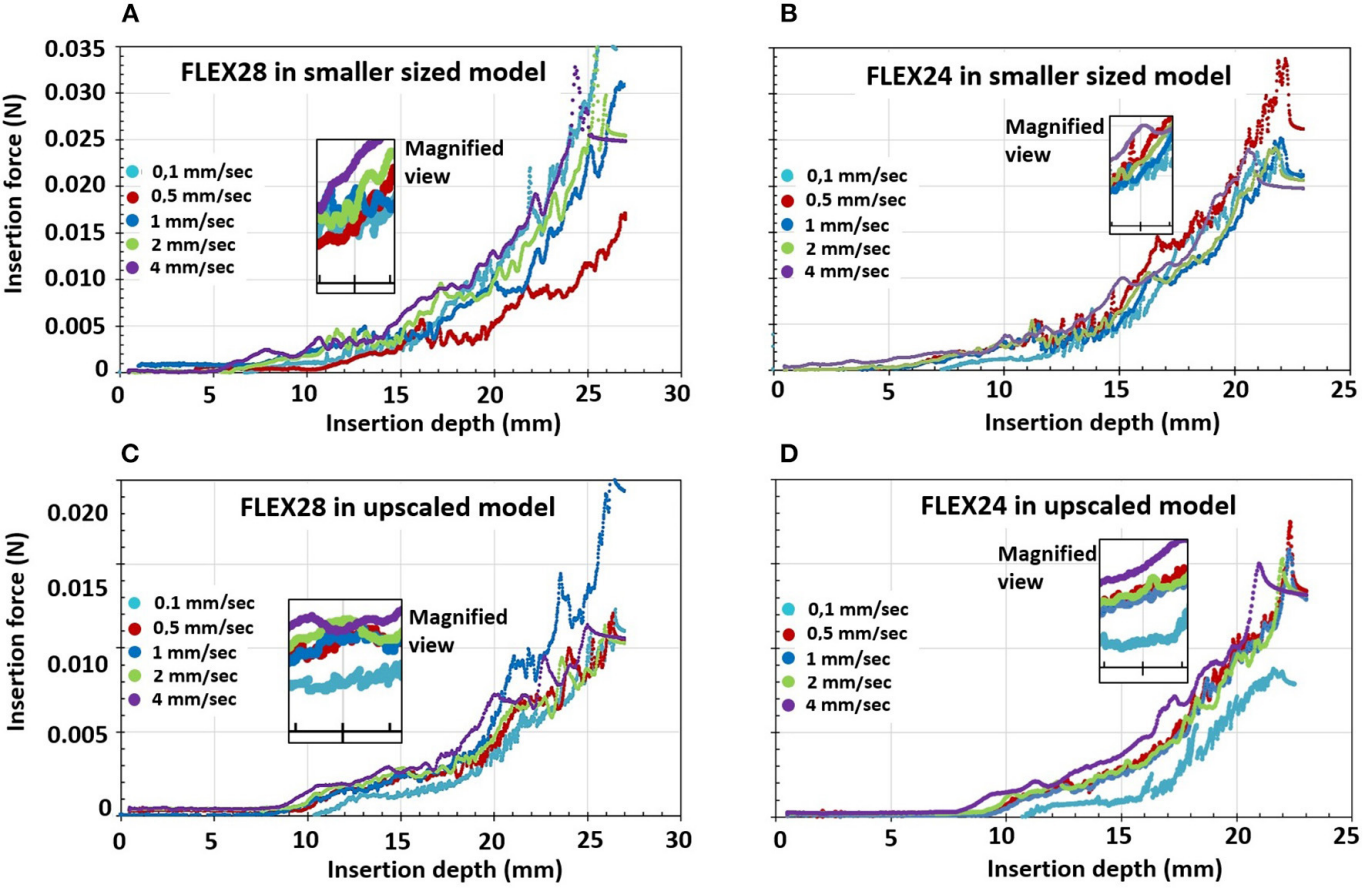
Electrode insertion force measurement of FLEX28 and FLEX24 electrodes in two different sized cochlear model applying five different insertion speeds of 0.1, 0.5, 1, 2, and 4 mm/s. **(A)** FLEX28 and **(B)** FLEX24 in the average sized cochlea model. **(C)** FLEX28 and **(D)** FLEX24 in the upscaled model. The inner magnified view shows the insertion force curves for various insertion speeds at 15 mm of insertion depth. The purple curve corresponds to the highest insertion speed of 4 mm/s showing higher insertion forces and the turquoise curve corresponds to the lowest insertion speed of 0.1 mm/s showing lower insertion forces.

Observation of insertion force in the first portion of the basal turn (at 15 mm of the insertion depth from the ST opening), indicated a trend for lower insertion forces at lower insertion speeds, whereby forces increased with higher insertion speeds (magnified view in Figure 6).

Overall, the insertion forces were lower in the upscaled model in comparison to the smaller sized ST model for both FLEX28 and FLEX24 electrodes at its full insertion depth, irrespective of the insertion speed, as shown in Figure 6. Two-way ANOVA test with replication showed a statistical significance of *p* < 0.0001 in insertion forces measured in smaller and upscaled ST models. Mean insertion forces for the insertion speeds are provided in Table 3.

**Table 3:** Mean insertion forces for FLEX28 and FLEX24 electrodes inserted at various insertion speeds in two different sized ST models.

**Electrode type**	**ST model**	**Mean insertion forces at insertion speeds (Newtons)**	**Significance**
FLEX28	Smaller model	0.025 (4 mm/s) 0.025 (2 mm/s) 0.03 (1 mm/s) 0.016 (0.5 mm/s) 0.034 (0.1 mm/s)	*p* < 0.0001
	Upscaled	0.01(4 mm/s) (2 mm/s) 0.02 (1 mm/s) (0.5 mm/s) 0.011 (0.1 mm/s)	
FLEX24	Smaller model	0.019 (4 mm/s) 0.02 (2 mm/s) 0.02 (1 mm/s) 0.026 (0.5 mm/s) 0.02 (0.1 mm/s)	*p* < 0.0001
	Upscaled	0.013 (4 mm/s) 0.013 (2 mm/s) 0.013 (1 mm/s) 0.013 (0.5 mm/s) 0.007 (0.1 mm/s)	

## Discussion

As we move toward personalized cochlear implantation, there is a clinical need to characterize patient specific anatomical variations. Specific areas of interest include, but are not limited to, (i) ST volume relative to basic cochlear parameters, (ii) electrode insertion force relative to insertion speeds dependent upon cochlear size, and (iii) the impact of insertion speeds on the insertion force at the S-value point. This pre-operative information will add to the wealth of information available to the surgeon to potentially influence intra-operative behavior. Furthermore, this information will be crucial to influence the development of robotic-assisted CI surgery (HEARO® system), controlled speed electrode insertion (ROBOTOL® system), and patient-specific pre-operative planning tools (OTOPLAN®).

This study has demonstrated that there are significant differences in the size of anatomically normal cochlea, in terms of ST volume and basic cochlear parameters. The range in ST volume in this study is similar to earlier reports measured from three *u*CT datasets (14). For the clinician, knowledge of the ST volume has specific clinical relevance. Firstly, choice of electrode array relative to cochlear duct length (CDL) and ST volume may be predictive of hearing preservation after CI, as demonstrated by Takahashi et al. (15). The authors elegantly demonstrated that bony cochlear volume [combined ST and scala vestibuli (SV)] was a predictive factor for hearing preservation following CI surgery. Furthermore, pre-operative appreciation of ST volume might assist in injection of pharmaceutical agents into the ST at the time of implantation. When an array is inserted, the equivalent volume of perilymph is displaced from the ST. Thus, appreciation of pre-operative ST volume and volume of the electrode array, will enable prediction of the necessary concentration of pharmaceutical agent to be injected.

However, ST volume cannot be measured in clinical pre-operative CT scans owing to their low resolution (~400 mm). Thus, a system which can predict ST volume is particularly valuable. In this study, the quasi-linear positive correlation between ST volume and A- and B-values enabled creation of a predictive algorithm to facilitate pre-operative prediction of ST volume on the basis of purely the A- and B-values as a novel finding. These two values are routinely measured pre-operatively as part of the radiological work-up prior to implantation. This approach is consistent with findings by Schurzig et al. who demonstrated that estimation of CDL is more accurate when considering both A- and B-values, rather than solely the A-value (16).

The second part of this study assessed the importance of individual cochlea size by focusing upon electrode insertion force in ST models of two different sizes/volumes. Results indicated that cochlear size, electrode array length and insertion speed are of varying importance to the force that the cochlea experiences during insertion. Insertion forces increased during insertion. The lower insertion forces in the upscaled model are likely owing to the greater cross-sectional dimensions of the ST model around the electrode array, thus offering less resistance. In clinical setup, larger ST volume would minimize the physical contact between the electrode array surface and the intra-cochlear structures including the basilar membrane. The other assumption is that with larger ST volume, the helicotrema would also be larger allowing the cochlear fluid to escape from the ST to the scala vestibuli. Earlier reports by Kontorinis et al. and Landry et al. indicated that higher insertion speed was associated with higher insertion forces (17, 18). Our findings were reflected in a study recently published by Aebischer et al. (19). The authors reported higher insertion forces with higher insertion speed across six different ST models (19). This was reflected in our study as well. The highest insertion speed of 4 mm/s recorded the highest insertion forces in both ST models and for both electrode arrays tested.

The first potential contact that the array may make with the cochlear lateral wall is at the S-value. When measured, the insertion force at the S-value was negligible. This may be related to the highly flexible nature of the FLEX electrodes utilized during this study. However, the forces start to increase after an insertion depth of approximately 10 mm and that's when the electrode start to bend inside the ST models. It is certainly of clinical interest to compare the insertion force at the S-value between arrays made of different materials from different CI brands. This could allow for adjustment of insertion speed at the S-value point depending on array material. Recently, Hendricks et al. studied the possibility of preventing the electrode tip touching the cochlear lateral wall, by magnetic guidance, by utilizing a modified electrode array with a magnet at the tip (20). All these research efforts work toward the aim of structure preservation during implantation to ensure hearing preservation and minimize the subsequent inflammatory process. Theoretically, it can be thought that the volume of the electrode array chosen for implantation in relation to the ST volume could as well be a deciding factor in the preservation of residual hearing as the electrode volume could restrict the vibrational properties of basilar membrane. This could be a study for the future on the evaluation of hearing preservation based on the electrode array length and volume chosen matching the ST volume estimated from the pre-operative cochlear parameters.

The ability to measure insertion forces during cochlear implantation is not feasible with manual clinician insertion; however, it may be incorporated into automated insertion devices. For instance, robotic insertion has the advantage of quantifiable haptic feedback, enabling direct feedback if insertion forces rise above a certain threshold. Our finding is in line with previous clinical reports on higher insertion forces and lower hearing preservation rates associated with higher insertion speed (21).

A wide variation in cochlear anatomy has been captured in this study of thirty μCT datasets. However, the variation in ST volume, A- and B-values resulted in a weaker correlation, which could be better defined by adding more datapoints. It would be more clinically relevant to compare insertion forces in cochleae of different sizes and shapes, with accurate RW reconstruction, rather than an upscaled model.

## Conclusions

ST volume was positively correlated with the A- and B-value, allowing the potential for pre-operative ST volume prediction from clinical CT scans. The S-value also increased linearly with the A-value. The ST size influenced electrode insertion force, in that in general higher insertion forces were observed with the smaller sized ST model compared to the upscaled model irrespective of the length of the implant. A trend toward lower insertion force for lower insertion speed and vice-versa was observed from the insertion force curves before the electrode reached its maximum insertion depth. Taken together these findings indicate that there are significant patient variations in cochlear size, and this may impact upon insertion forces. Whilst insertion forces increase during advancement of the CI, the absolute value may be difficult to sense manually (measured in milli-newtons). Such small changes in force would be best measured by automated insertion systems, which employ more accurate haptic feedback than is possible by the human hand.

## Data Availability Statement

Half of the data were the result of HearEU project, https://cordis.europa.eu/project/id/304857. The data can be obtained by asking the project co-ordinator. The remaining raw data supporting the conclusions of this article will be made available by the authors, without undue reservation.

## Author Contributions

AD: study design, data collection, data analysis, figure preparation, manuscript writing, and literature search. CS: study design, data collection, data analysis, and manuscript writing. MB and PV: critical review, data analysis, and manuscript review. VV: critical review, literature search, and manuscript review. All authors contributed to the article and approved the submitted version.

## Conflict of Interest

AD is employed at MED-EL in scientific roles with no marketing activities. The remaining authors declare that the research was conducted in the absence of any commercial or financial relationships that could be construed as a potential conflict of interest.

## Publisher's Note

All claims expressed in this article are solely those of the authors and do not necessarily represent those of their affiliated organizations, or those of the publisher, the editors and the reviewers. Any product that may be evaluated in this article, or claim that may be made by its manufacturer, is not guaranteed or endorsed by the publisher.
