# Corrigendum: Cochlear Size Assessment Predicts Scala Tympani Volume and Electrode Insertion Force- Implications in Robotic Assisted Cochlear Implant Surgery

**DOI:** 10.3389/fsurg.2021.789184

**Published:** 2021-10-27

**Authors:** Anandhan Dhanasingh, Chloe Swords, Manohar Bance, Vincent Van Rompaey, Paul Van de Heyning

**Affiliations:** ^1^Research and Development Department, MED-EL, Innsbruck, Austria; ^2^Department of Translational Neurosciences, Faculty of Medicine and Health Sciences, University of Antwerp, Antwerp, Belgium; ^3^Department of Physiology, Development and Neurosciences, University of Cambridge, Cambridge, United Kingdom; ^4^Department of Clinical Neurosciences, University of Cambridge, Cambridge, United Kingdom; ^5^Department of Otorhinolaryngology and Head & Neck Surgery, Antwerp University Hospital, Antwerp, Belgium

**Keywords:** scala tympani volume, cochlear size, electrode insertion speed, electrode insertion force, robot assisted surgery

In the original article, there was a mistake in Figure 6 as published. The horizontal axes of Figures 6B,D should extend between 0 and 25 mm, not 0 and 30 mm as given in the original, incorrect version. The corrected Figure 6 appears below. Additionally, values in the figure were originally presented using decimal commas; the updated figure now uses decimal points, for consistency with the text of the article.

The values in the original Figure 4 were also presented using decimal commas; this has also been corrected. The new Figure 4, provided below, contains decimal points, for consistency with the text of the article.

**Figure 4:**
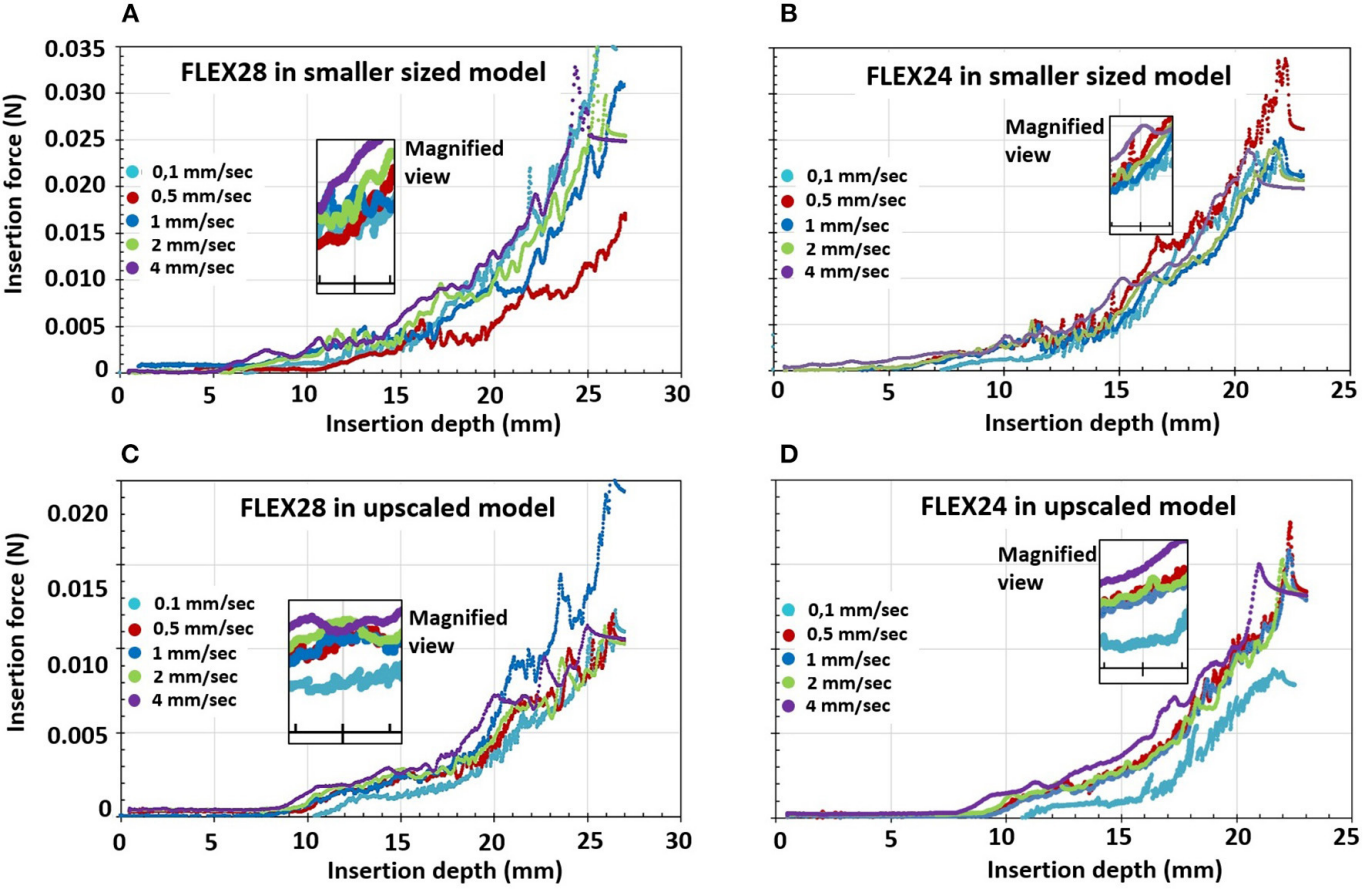
Scatter plots comparing **(A)** A-value and ST volume; **(B)** B-value and ST volume; **(C)** A- and B- values; and **(D)** A- and S-values.

**Figure 6:**
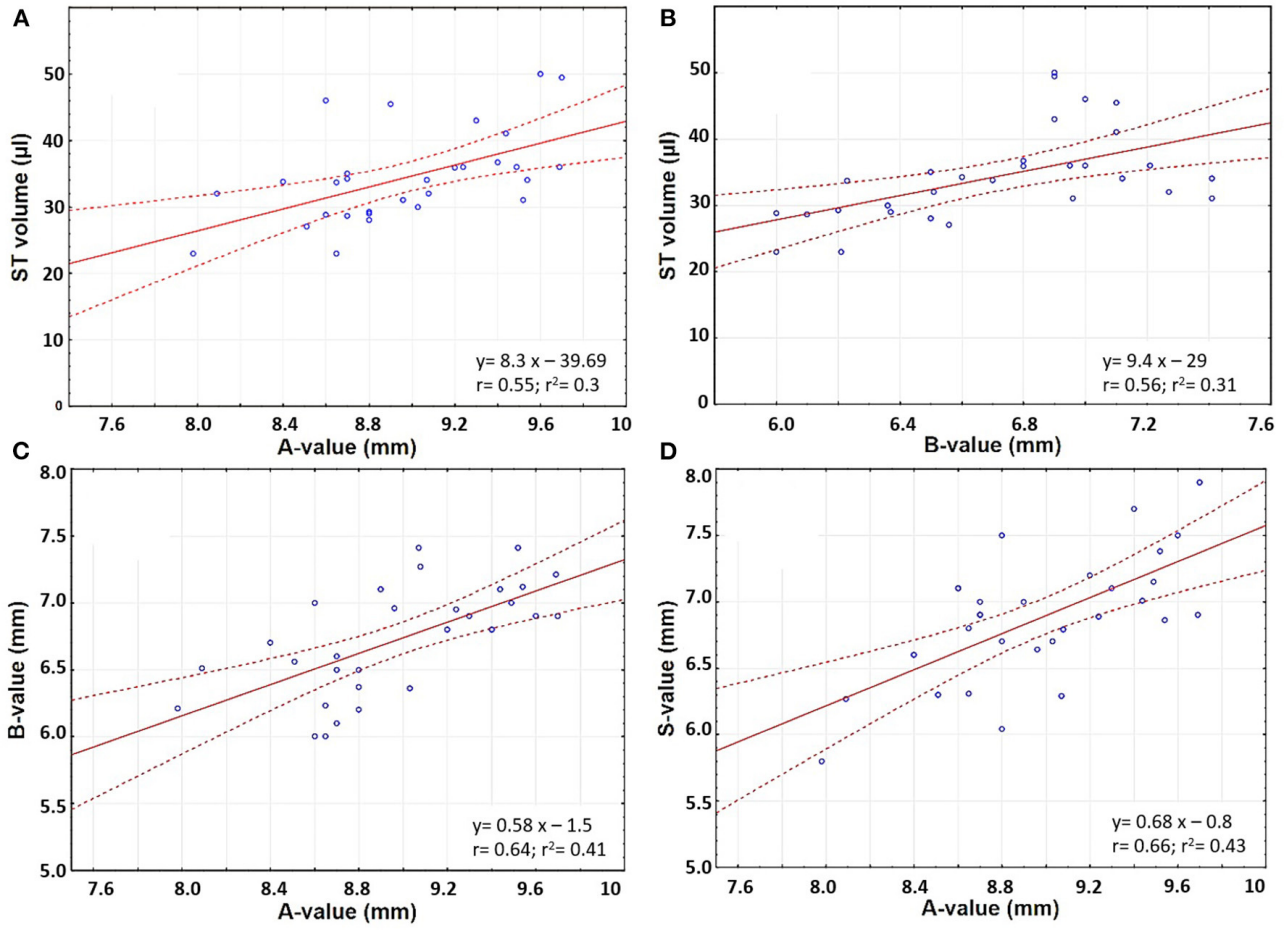
Electrode insertion force measurement of FLEX28 and FLEX24 electrodes in two different sized cochlear model applying five different insertion speeds of 0.1, 0.5, 1, 2, and 4 mm/s. **(A)** FLEX28 and **(B)** FLEX24 in the average sized cochlea model. **(C)** FLEX28 and **(D)** FLEX24 in the upscaled model. The inner magnified view shows the insertion force curves for various insertion speeds at 15 mm of insertion depth. The purple curve corresponds to the highest insertion speed of 4 mm/s showing higher insertion forces and the turquoise curve corresponds to the lowest insertion speed of 0.1 mm/s showing lower insertion forces.

The authors apologize for these errors and state that this does not change the scientific conclusions of the article in any way. The original article has been updated.

## Publisher's Note

All claims expressed in this article are solely those of the authors and do not necessarily represent those of their affiliated organizations, or those of the publisher, the editors and the reviewers. Any product that may be evaluated in this article, or claim that may be made by its manufacturer, is not guaranteed or endorsed by the publisher.

